# The relationship of single-family detached house prices with neighborhood walkability and disaster risk

**DOI:** 10.1371/journal.pone.0292657

**Published:** 2023-10-11

**Authors:** Hongjik Kim, Hiroki Baba, Chihiro Shimizu, Kimihiro Hino

**Affiliations:** 1 Department of Urban Engineering, Graduate School of Engineering, The University of Tokyo, Tokyo, Japan; 2 Center for the Promotion of Social Data Science Education and Research, Hitotsubashi University, Tokyo, Japan; Institute for Advanced Sustainability Studies, GERMANY

## Abstract

People’s preferences regarding their neighborhood environment can vary depending on their socioeconomic status and the cities where they live. This study aims to discern the relationship between neighborhood environment factors and single-family detached house sales by sale price and by central and noncentral cities. We analyzed sale prices in the Tokyo Metropolitan Area from 2015 to 2020. The neighborhood environment was assessed using flood/sediment risk and neighborhood walkability measured by net residential density, intersection density, and facility density (walking opportunity). Flood and sediment risk is a major concern that restricts the available land and is included as a negative aspect of the neighborhood environment, taking the topographic features into consideration. A comparison of the results showed that the preference for neighborhood walkability varies by socioeconomic status as well as by target cities. For most facility types, the number of walking opportunities within walking distance from houses was found to be positively related to the sale price of single-family detached houses in all quantiles. The relationship of house price with population and intersection density was found to vary depending on the price level, with a negative relationship with the sale price of relatively more expensive houses being exhibited. People who considered buying houses with relatively higher sale prices were found to devalue houses located in flood/sediment-hazardous areas more. However, it was also found that the negative relationship was slightly mitigated in the highest quantile of sale prices for houses in areas with a moderate flood risk (maximum flooding depth: 3–5 m). Plains near rivers with amenities offer high walkability but pose a flood risk, resulting in a trade-off between flood risk and neighborhood walkability. The findings suggest the use of indices representing diverse preferences in accordance with the target socioeconomic status when policymakers assess the neighborhood environment.

## Introduction

Urban agglomeration has proceeded through the gathering of firms and workers in cities, alongside urban growth [1–3]. Urban agglomeration creates clusters of amenities that hinge on the role of cities as centers of consumption [4]. Meanwhile, urban agglomeration brings negative externalities, such as congestion and crime, which decrease residents’ quality of life [5]. Suburban residential areas have been developed with population growth and decreased transportation costs owing to the use of cars and public transportation [6, 7]. This has created metropolitan areas, containing central cities, which are defined as cities with a large population and a high level of urban agglomeration. Conversely, population aging and decline, which many countries are facing or anticipated to face [8], can strengthen the agglomeration of places where people perform daily activities [9]. Therefore, the preference for urban agglomeration and the neighborhood environment is an important aspect of residential choice [10] and the sustainable development of neighborhoods where people desire to live.

Areas with clusters of amenities have high neighborhood walkability, which refers to the proximity to amenities (or walking opportunities) [11]; a walkable neighborhood promotes residents’ quality of life [12] and increases its property values [13, 14]. Walkability is defined as built environment conditions that encourage walking behavior [15, 16]. Neighborhood walkability is commonly measured by the “5Ds” [17]: *density*, *diversity*, *design*, *destination accessibility*, and *distance to transit* [18–20]. Areas with high neighborhood walkability refer to those with a large population (*density*), high street connectivity (*design*), and good access to diverse facilities (*diversity* and *destination accessibility*) and public transportation (*distance to transit*). A close distance from homes to amenities (measured by facility density within an area, for example) is the most important factor of neighborhood walkability that can be generalizable regardless of the urban structure [11]. Areas with high street connectivity and easy access to public transportation can further facilitate the access of pedestrians living in those areas to facilities by reducing their travel time to reach their destinations [19, 20]. The presence of a large population in an area creates a greater demand for diverse amenities, thereby ensuring their availability in the area [4].

Urban agglomeration is constrained by topographic features; therefore, people tend to live on plains, which have high walkability owing to the agglomeration [21]. Meanwhile, cities with restricted available land due to geographic constraints sometimes suffer from natural disasters [22]. Among such natural disasters, floods and sediments affect a local area and depend on topographic features (i.e., plains near rivers have a flood risk and hilly areas have a sediment risk; therefore, the flood/sediment risk varies depending on the location within a city). For example, cities in Japan have restricted available land because they are located on an island with many mountains close to the sea and rivers. There are few plains in cities with geographic constraints, and their flood depth is expected to be high, particularly in the case of plains near rivers. Plains with high walkability attract many people to reside on them and create central cities with a large population and a high level of urban agglomeration [4]; however, they also expose them to expected flood disasters. Therefore, hilly areas have been developed as residential areas to accommodate the increasing population [23]. The newly developed residences in hilly areas have a high sediment disaster risk and are relatively nonwalkable owing to the slopes. Mobility tends to decrease as people age [24], and the proximity to amenities is especially important for people with limited mobility [11, 12]. However, there are few walking opportunities in hilly areas; thus, the cluster of amenities tends to be located on plains [21]. The slopes in hilly areas can be a burden when walking to the cluster of amenities on plains, especially for people with limited mobility; therefore, slopes discourage walking among people living in such areas [25, 26]. Vulnerability to disaster risk also increases in such areas due to the functional decline [27]. This can make people in countries facing population aging (such as Japan) place more importance on the amenities close to their homes, thereby strengthening urban agglomeration on plains [9]. Consequently, there may be a trade-off between flood risk and neighborhood walkability. Furthermore, in hilly areas with a sediment risk and steep slopes, residents in aging cities may opt for plains with higher walkability. The neighborhood walkability and flood/sediment risk represent the positive and negative aspects of the neighborhood environment, taking the topographic features into consideration. Earthquakes and typhoons are natural disturbances that exert impacts across cities, and they trigger floods and sediments [28–30]. In addition, the prospect of a warmer climate is projected to increase the global flood/sediment risk [31, 32]. Therefore, we focus on the flood/sediment risk as a parallel to neighborhood walkability.

As previously mentioned, plains with high walkability attract people [4]. Assuming constraints in the available land and housing supply, the prices of houses located in safe and walkable neighborhoods (as well as their rents and the land prices) will increase due to a shift in the aggregate demand corresponding to the attractiveness. Given that the sale prices of houses are the result of individuals’ residential choice behavior, they can be considered a monetary indicator of their revealed preference [33–35]. By controlling for houses’ characteristics, it is possible to use the sale price to estimate which neighborhood characteristics people value when choosing a residential area where they desire to live [35]; this is known as the “hedonic price approach.” This suggests that an analysis using the sale price of houses enables us to clarify individuals’ preference for neighborhood characteristics, taking into consideration the relationship between neighborhood walkability and flood/sediment risk.

Many empirical studies have shown that being located in areas with a flood/sediment risk is negatively related to the sale price of houses [36–41]; conversely, a positive relationship has been observed between the neighborhood walkability and the sale price of houses [13, 14, 42–46]. Studies on the relationship between walkability and house price have reported that a high population density and a close distance to amenities and to public transportation are positively related to the sale price of houses [42–46]. With regard to floods, studies conducted in the United Kingdom [36] and Taiwan [40] have reported a positive relationship between house prices and proximity to rivers, as well as a negative relationship between house prices and being located in a flood-hazardous area. On the other hand, some studies reported a negative relationship between population density and house price, depending on the target area [47–49]. Most of these studies have focused on the difference in the relationship within the geographical context: comparing the relationship by using models considering geographical dependencies [42–44, 46, 47] or by analyzing for each subregional group [45, 48, 49]; few studies have considered the difference in the relationship based on home buyers’ income levels. The way in which people perceive the same neighborhood environment may vary due to differences in their consumption and lifestyle, depending on their residential location and current socioeconomic status [50, 51]; the relationship between house prices and neighborhood characteristics may also vary based on the difference in their perception. People who live in central cities or earn a high income may place more importance on a safe and walkable neighborhood [52–54]; therefore, the neighborhood walkability and flood/sediment risk can exert a greater impact on the sale price of the houses that they live in or consider buying. A comparison by income level and by centrality in the relationship of house prices with neighborhood walkability and flood/sediment risk will provide clues for understanding the neighborhood where people desire to live in the context of the possible difference in individuals’ perception of neighborhood characteristics. A detailed explanation of the possible differences in the perception of neighborhood walkability and flood/sediment risk follows.

Individuals’ preference for neighborhood characteristics can vary due to their car-dependent lifestyle [55, 56] and budget flexibility/constraints [52–54]. In the case of neighborhood walkability, people living in suburban areas may value good access (within walking distance) to amenities and public transportation from their homes less than those living in central cities because they are more likely to prefer car-oriented travel [55, 56]. Conversely, high-income earners can afford to pay for services to maximize their utility; thus, they may prefer to have many amenities and a high level of services within walking distance of their home. Indeed, creative people are more likely to prefer walkable neighborhoods [52, 53]. In contrast, low-income earners may prefer affordable services. Flood/sediment risk, as previously mentioned, can be considered a counterpart to neighborhood walkability due to the constraints of plains without risks. There are few plains with a low flood risk in the suburbs, so more people living in such areas may opt to live in a hilly area owing to their weaker preference for neighborhood walkability (due to their car-dependent lifestyle) than those living in central cities [23]. This possibly slightly lowers the sale price of houses located in areas with a flood/sediment risk. Furthermore, due to the increases in the sale price of houses in safe and walkable areas, low-income earners generally do not consider buying houses in those areas [54]. In other words, low-income earners may not be able to pay to avoid the risks or to lower the sale price to compensate for the risks. Therefore, the relationship between sale price and both neighborhood walkability and flood/sediment risk should be examined, taking into consideration the possible difference in perception according to income level and central and noncentral cities.

In summary, this study aims to discern differences in the relationships between neighborhood characteristics and single-family detached house sale prices by price level as well as by cities’ central or noncentral status. We focus on the sale prices of single-family detached houses rather than condominiums. Condominiums have common places containing amenities within buildings or complexes owing to their large population, but single-family detached houses do not (i.e., neighborhood walkability is more important for single-family detached houses than for condominiums). In addition, flood/sediment risk is more important for single-family detached houses than for condominiums due to their lower number of stories (single-family detached houses tend to have two or three floors) [57]. The land price includes commercial/business land use, which is more likely to be found in areas with high walkability, not only residential use. For housing rents, because single people are the primary tenants in Japan and tend to stay for shorter periods of time [58, 59], they may not find accommodation and live in the neighborhood where they desire to reside the most (i.e., the housing rents may not be the most reflective of residents’ preference). The wide variety of locations of single-family detached houses enables us to clarify the differences in residents’ revealed preference (based on their sale price), taking into consideration the relationship between neighborhood walkability and flood/sediment risk. High-income households are more likely to have a high demand for houses [60], resulting in increased house sale prices [43, 61–63]. Assuming a budget constraint, those who can pay more for a house with similar features to others are more likely to be high-income earners. Therefore, an analysis considering differences in sale prices provides clues to how low- and high-income earners perceive the neighborhood environment and enables us to determine the difference in their preferences. It is hypothesized that neighborhood walkability and flood/sediment risk exert a greater impact on the sale price of houses located in central cities and those with a higher price range.

## Materials and methods

### Data sources

This study analyzed the sale prices of single-family detached houses sold in the Tokyo Metropolitan Area (Tokyo, Saitama, Chiba, and Kanagawa prefectures) from 2015 to 2020. The sale price data were collected by Recruit Co., Ltd., which is one of the largest agents of residential property information in Japan. The data included both new and pre-owned single-family detached houses. In total, 136,672 single-family detached houses were sold between 2015 and 2020. Of these, some houses were omitted owing to missing values regarding floor space (65,383 samples; on average, they were more likely to have a larger land area [124.53 m^2^], and be located close to both the nearest station [10.74 min] and Tokyo Station [39.57 min] than the selected sample [113.22 m^2^, 13.04 min, 44.72 min, respectively]). Consequently, the analytical sample consisted of 71,289 samples. The sale price data from an agent were among all the transactions in the Tokyo Metropolitan Area; however, there was variety in the areas of the samples, ranging from urban to suburban and rural areas. Therefore, these data can be used to capture trends regarding the sale market of single-family detached houses in a metropolitan area.

**Fig 1:**
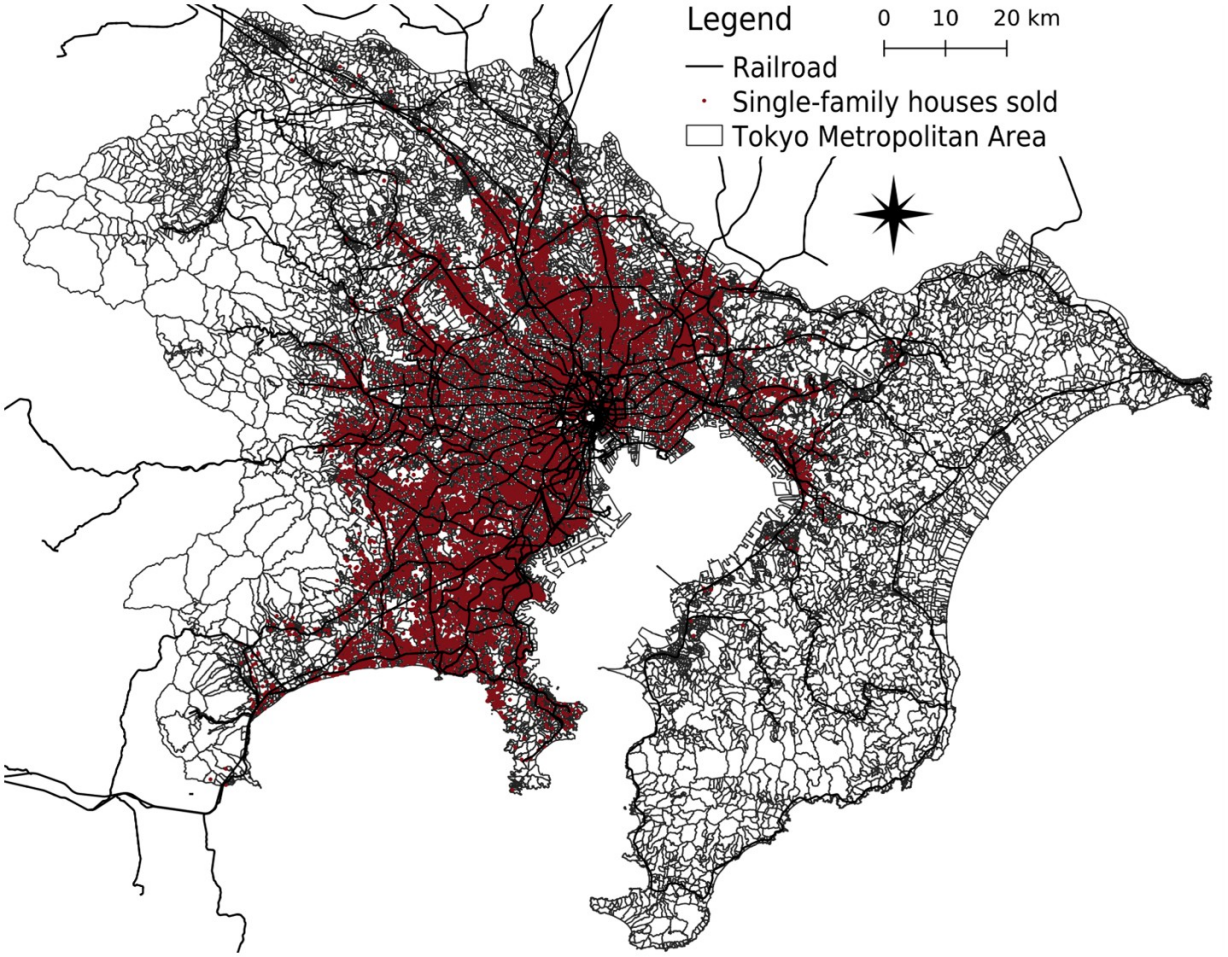
Location of the analytic sample (single-family detached houses sold between 2015 and 2020) and its geographical relationship with the Tokyo Metropolitan Area.

Census data drawn from the Statistics Bureau of Japan in 2015 were linked to the data on the sale prices of single-family detached houses sold. There were 4,126 census tracts in the areas where the analytic sample was located. Because the samples comprised single-family detached houses sold, they were more likely to be located in areas alongside railways, where many residents reside in the Tokyo Metropolitan Area (Fig 1). Furthermore, facility data, taken from telephone directory data with associated location information (Telepoint Pack! published by Zenrin Co. Ltd.) in 2015, along with location data of bus stops in 2010 and schools in 2013 from the Ministry of Land, Infrastructure, Transport, and Tourism, were linked to the data used to assess walking opportunities, that is, amenities within a walkable distance from each house. The Advanced Digital Road Map Database 2015 (Sumitomo Electric Industries, Ltd.) was used to consider network distance and street connectivity. Hazard maps in 2019 regarding flood and sediment disasters were obtained from the Ministry of Land, Infrastructure, Transport, and Tourism and linked to the data to determine whether each house was located in a hazardous area. Flood hazard areas have been designated by national and local governments as areas that are expected to be inundated when rivers overflow because of the maximum projected rainfall [64]. Furthermore, the ministry has designated hazardous sediment areas based on surveys of topography, geology, and land use [64]; the provision of hazard information (e.g., hazard maps) is mandatory before transactions take place [65].

### Variable descriptions

Table 1 shows the characteristics of single-family detached houses sold between 2015 and 2020. The sale price was recorded for each single-family detached house sold. The characteristics of each house, such as years/months of transactions, floor space, land area, age of house, road width in front of the house, and main window orientation, were also recorded and served as control variables. Years/months of transactions were included on a quarterly basis to account for the impact of temporal factors on sale price (e.g., inflation). The maximum flooding depth of the area where each house was located was assessed and the results were classified into four groups [66] according to the risk of damage to the floors, ranging from no risk (0 m of flooding depth) to high risk (5 m and higher of flooding depth, where the water level on the second floor becomes hazardous). In the case of a sediment disaster, we identified whether each house was inside a hazardous sediment area; this was defined as a binary variable. Land use zones and travel time to Tokyo Station from the nearest station were included to account for designated zoning restrictions on building height and building types for the area where each house is located and the distance to the central business district, respectively.

**Table 1:** Characteristics^a^ of single-family detached houses sold between 2015 and 2020.

	Target area	Central cities	Other cities
Sale price (JPY10,000)	4,529 ± 3,221	5,314 ± 3,797	3,487 ± 1,764
Year of transactions (month is omitted)			
2015	9,082 (12.7)	5,357 (13.2)	3,725 (12.2)
2016	8,841 (12.4)	5,010 (12.3)	3,831 (12.5)
2017	9,032 (12.7)	5,089 (12.5)	3,943 (12.9)
2018	13,603 (19.1)	7,640 (18.8)	5,963 (19.5)
2019	15,438 (21.7)	8,845 (21.8)	6,593 (21.5)
2020	15,293 (21.5)	8,720 (21.4)	6,573 (21.5)
Floor space (m^2^)	106.47 ± 38.42	108.22 ± 41.90	104.14 ± 33.10
Land area (m^2^)	113.22 ± 52.19	107.79 ± 54.18	120.44 ± 48.49
Road width in front of houses (m)	5.38 ± 2.48	5.30 ± 2.42	5.48 ± 2.55
Age of houses (months)	179.9 ± 101.07	187.4 ± 101.97	170.0 ± 99.00
Main window orientation			
Facing south	12,626 (17.7)	6,949 (17.1)	5,677 (18.5)
Other	58,663 (82.3)	33,712 (82.9)	24,951 (81.5)
Travel time to Tokyo Station from the nearest station in daytime (min)	44.72 ± 13.09	39.98 ± 12.18	51.00 ± 11.51
Maximum flooding depth (m)			
0.0	56,440 (79.2)	32,653 (80.3)	23,787 (77.7)
(0.0, 3.0)	9,287 (13.0)	4,410 (10.8)	4,877 (15.9)
[3.0, 5.0)	4,692 (6.6)	3,176 (7.8)	1,516 (4.9)
[5.0, inf]	870 (1.2)	422 (1.0)	448 (1.5)
Sediment-hazardous area			
Houses inside the area	2,107 (3.0)	1,256 (3.1)	851 (2.8)
Houses outside the area	69,182 (97.0)	39,405 (96.9)	29,777 (97.2)
Land use zones			
Exclusively low-rise residential zone	29,194 (41.0)	16,469 (40.5)	12,725 (41.5)
Mid/high-rise-oriented residential zone	15,112 (21.2)	8,492 (20.9)	6,620 (21.6)
Residential zone	14,341 (20.1)	8,925 (21.9)	5,416 (17.7)
Commercial zone	3,614 (5.1)	2,523 (6.2)	1,091 (35.6)
No land use zone designated	3,868 (5.4)	1,377 (3.4)	2,491 (8.1)
Sample number	71,289	40,661	30,628

^a^ The mean and standard deviation (SD) are displayed for the continuous variables (and the number of samples for the binary and categorical variables).

The neighborhood walkability—net residential density, intersection density, walking time to the nearest station, and facility density—was assessed for each single-family detached house (Table 2). Net residential density, intersection density, and facility density are popular indicators used to measure neighborhood walkability in many countries [67–71]. An area with high net residential density is considered to be one that is densely developed. Net residential density was measured at the level of the census district where each single-family detached house was located. Facility density was measured as the density of each facility type within a catchment area of 1,200 m from each house along a road network. The distance of 1,200 m corresponds to a 15-minute walk, which is the average walking distance to neighborhood destinations [72]. The density of neighborhood facilities enabled us to assess the number of destinations that residents can travel to within a walkable distance. Witten et al. [71], for example, considered eight types of facilities (education, transport, recreation, social and cultural, food retail, financial, health, and other retail). Similarly to the classification by Witten et al. [71] (but with more detailed facility types), we considered 16 types of facilities, including cafés, restaurants, gyms, hobby classes, schools, hospitals, train stations, bus stops, and cultural institutions (museums, libraries, community centers, etc.). These facilities provide leisure, recreation, and welfare opportunities (e.g., schools can also be perceived as recreation facilities by making their playgrounds available as public places for recreation activities), which are considered attractive components of the neighborhood environment and are major destinations in daily living [56, 71]. Thus, a high density of neighborhood facilities indicates more neighborhood walking opportunities.

**Table 2:** Neighborhood characteristics^a^ of single-family detached houses sold between 2015 and 2020.

	Target area	Central cities	Other cities
Net residential density^b^	1,918 ± 1,887	2,250 ± 2,137	1,477 ± 1,374
Intersection density (four-way)	53.5 ± 25.46	61.50 ± 26.94	42.94 ± 18.68
Walking time to the nearest station (min)	13.04 ± 5.75	12.29 ± 5.66	14.03 ± 5.72
Facility density			
Bars	13.46 ± 22.15	16.64 ± 26.62	9.23 ± 13.03
Cafés	2.73 ± 4.67	3.54 ± 5.65	1.65 ± 2.51
Restaurants	25.27 ± 32.49	31.71 ± 39.39	16.72 ± 16.39
Hobby classes	4.24 ± 4.55	4.98 ± 4.91	3.27 ± 3.81
Cultural institutions	3.58 ± 4.34	4.23 ± 5.16	2.71 ± 2.68
Parks	0.15 ± 0.34	0.21 ± 0.38	0.09 ± 0.27
Gyms and fitness clubs	2.75 ± 2.61	3.19 ± 2.95	2.16 ± 1.93
Convenience stores	4.31 ± 3.26	5.27 ± 3.77	3.04 ± 1.75
Schools	2.13 ± 1.32	2.55 ± 1.45	1.57 ± 0.86
Childcare facilities	4.73 ± 2.67	5.64 ± 2.80	3.52 ± 1.89
Retail shops	5.28 ± 10.30	6.52 ± 12.01	3.64 ± 7.12
Welfare facilities for older adults	8.32 ± 4.72	9.99 ± 4.84	6.11 ± 3.48
Hospitals	0.24 ± 0.61	0.30 ± 0.66	0.16 ± 0.51
Pharmacies	6.15 ± 4.45	7.35 ± 4.77	4.56 ± 3.38
Bus stops	7.39 ± 2.94	7.35 ± 2.54	7.43 ± 3.40
Stations	0.47 ± 0.64	0.59 ± 0.72	0.30 ± 0.45
Sample number	71,289	40,661	30,628

^a^ The mean and standard deviation (SD) are displayed for the neighborhood characteristics. All the densities are estimated per square kilometer.

^b^ Net residential density is estimated for each census tract where the targeted samples are located (4,126 census tracts); that is, buffered areas based on a 1,200 m network distance away from houses are used in the case of intersection and facility density.

Stations were also assessed using the walking time to the nearest station from each house based on the average walking speed. Because we included both the distance and the density of stations, it was possible to consider not only the proximity to the nearest station from residents’ homes but also their options to access a station (e.g., when there are many stations, people who visit a destination within their neighborhood can more easily access the nearest station from that point). In addition, the proximity to the nearest station enabled us to consider each house’s proximity to a local central area (i.e., the Tokyo Metropolitan Area was developed alongside railways, and areas near stations were considered to be local central areas). With regard to intersection density, we selected four-way intersections (20.5% of all intersections) because they represent good neighborhood walkability and frequent use of public transportation better than three-way intersections [73–75]; their density was measured in the same way as facility density. High intersection density indicates that the road network near each house is more likely to be a grid pattern.

### Analytic method

A conditional quantile regression model was used to investigate the relationships between the neighborhood characteristics and the conditional quantiles of the sale price rather than the mean value [76–78]. Given that the quantiles were conditional, the model could consider the sale price after controlling for other factors, such as the floor space and age of the house, which enabled us to test the relationships between sale price and neighborhood characteristics. As previously mentioned, assuming a budget constraint, those who can pay more for a house with similar features to others are more likely to be high-income earners. In other words, the difference by conditional quantiles in the relationship between the sale price and the neighborhood characteristics indicates the difference by income level in home buyers’ revealed preference for the neighborhood characteristics. The sale price was included in the model as a natural logarithmic scale owing to the large variance. The net residential density, intersection density, and facility density were transformed into a nonnegative form, *ln* (1 + *density*), which is a special case of Box–Cox transformation; this enabled us to escape a negative infinity issue (i.e., zero values) and transform the right-skewed distribution to make it proximal to a normal one [79]. The years/quarters of transactions, floor space, land area, age of the houses, road width in front of the houses, main window orientation, land use zones, and travel time to Tokyo Station from the nearest station were adjusted. The models were estimated in accordance with conditional quantiles ranging from the 0.05 to the 0.95 quantile at 0.05-point intervals. The 0.50 quantile corresponds to the median.

We estimated two models as follows:

*Model 1*: *Regression model considering differences by centrality*
*Model 2*: *Regression model considering differences by income level*

**Fig 2:**
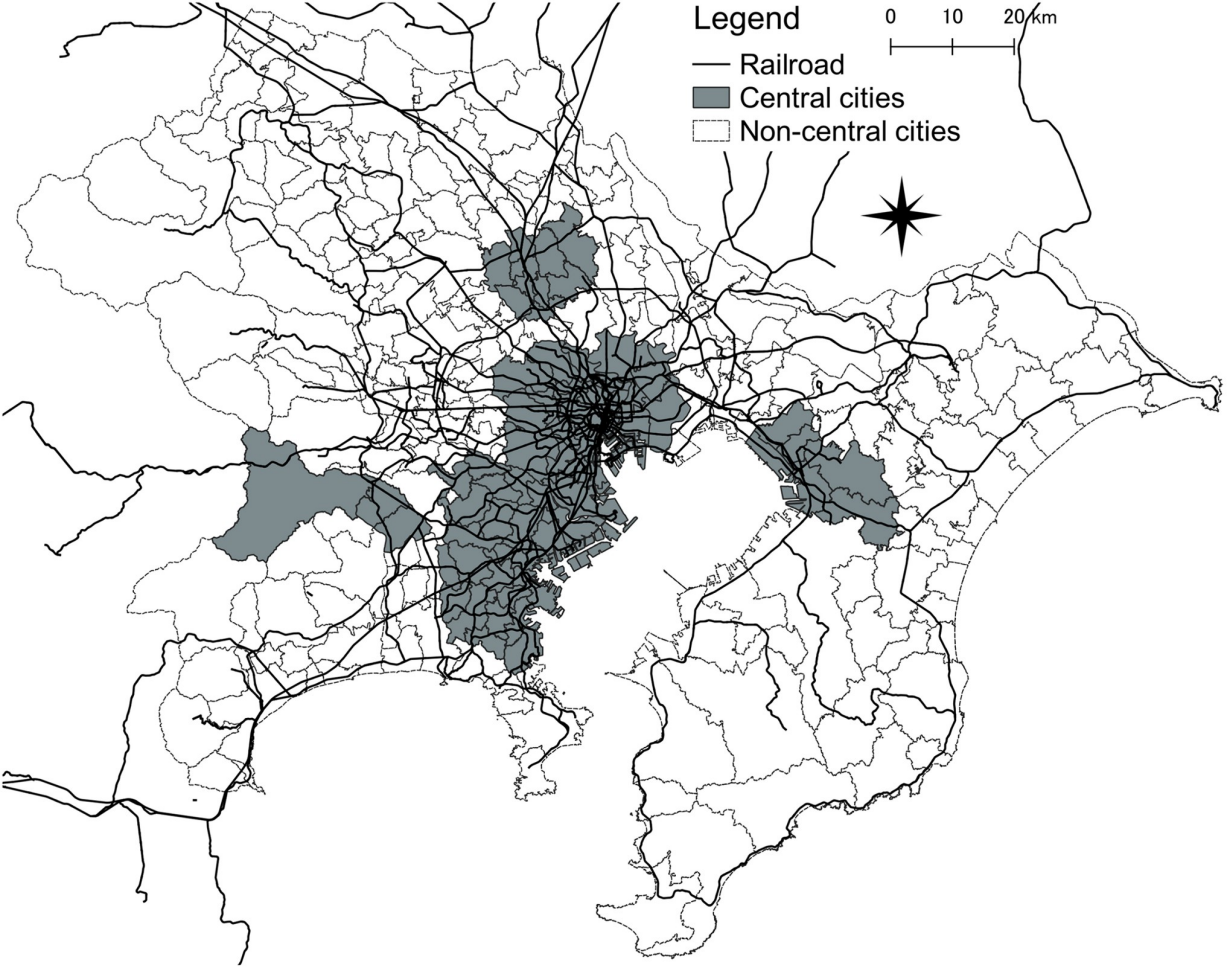
Locations of the central and noncentral cities.

The analysis was conducted in three steps. We initially estimated the conditional quantile regression model (Model 1) to capture the relationships regarding the median of the sale price, using the total samples as well as those of subareas grouped by their centrality: central cities (the 23 special wards of Tokyo and the cities of Kawasaki, Yokohama, Chiba, Saitama, and Sagamihara, which are the main cities in the Tokyo Metropolitan Area; Fig 2) and other cities. The 23 special wards of Tokyo are the most populated and densely developed areas in Japan. The five cities are known as “government ordinance designated cities” (generally referred to as “big cities” in Japan), which are designated owing to the large number of people who live in and commute to them [80], indicating a high level of urban agglomeration. Because Japan is an island with many mountains close to the sea and rivers, even the central cities include sediment-hazardous areas, and some houses are inside such areas (Table 1). We also briefly compared the coefficients from the model regarding the median sale price (for the total sample) with those from the models (for 0.15 and 0.85 conditional quantiles of the sale price; Model 2). The results of the models by subareas (Model 1) and by representative conditional quantiles (Model 2) enabled us to clarify the differences in the relationships between the neighborhood characteristics and the sale price of single-family detached houses by centrality and price level. Finally, we explored and visualized the differences among the conditional quantiles (from 0.05 to 0.95 at 0.05-point intervals) of the sale price and their relationships with neighborhood characteristics. Based on a comparison of each neighborhood characteristic, we discerned the relationship between neighborhood environment and conditional quantiles of single-family detached house prices.

## Results

### Results of regression models by centrality

Table 3 summarizes the results of the conditional quantile regression models (for the median sale price, which is the 0.50 conditional quantile; Model 1) for all samples and subareas (central and other cities). Column 3(a) shows the results for all samples from the target area. High net residential density was negatively related to the median sale price of single-family detached houses (β = -0.003). Variables related to flood and sediment risk also showed negative relationships (β = -0.122, β = -0.188, and β = -0.219 for flood risk by maximum flooding depth; β = -0.026 for sediment risk). In terms of facility density, the density of most facility types was positively related to the median sale price, except for bars and cultural institutions (β = -0.077 and β = -0.060, respectively). A shorter time distance to the nearest station was negatively related to the median sale price (β = -0.011). The intersection density was found to be insignificant.

**Table 3:** Comparison of the results^a^^,^^b^ of the conditional quantile regression models (50% quantile) between central and other cities.

	(a) Target area	(b) Central cities	(c) Other cities	Difference in the direction of the coefficient^d^ (a) → (b) → (c)
Higher net residential density^c^	-0.003 (0.001) ***	-0.010 (0.001) ***	-0.004 (0.001) **	− → − → −
Higher intersection density^c^	-0.006 (0.003)	0.001 (0.004)	-0.099 (0.004) ***	· → · → −
Shorter time distance to the nearest station	-0.013 (0.000) ***	-0.015 (0.000) ***	-0.009 (0.000) ***	− → − → −
Higher facility density^c^				
Bars	-0.077 (0.003) ***	-0.086 (0.004) ***	-0.049 (0.003) ***	− → − → −
Cafés	0.104 (0.003) ***	0.095 (0.004) ***	0.104 (0.004) ***	+ → + → +
Restaurants	0.014 (0.004) ***	0.048 (0.006) ***	-0.006 (0.004)	+ → + → ·
Hobby classes	0.032 (0.003) ***	0.074 (0.004) ***	0.018 (0.004) ***	+ → + → +
Cultural institutions	-0.060 (0.003) ***	-0.050 (0.004) ***	-0.029 (0.004) ***	− → − → −
Parks	0.052 (0.005) ***	0.023 (0.005) ***	0.043 (0.011) ***	+ → + → +
Gyms and fitness clubs	0.063 (0.003) ***	0.070 (0.003) ***	0.045 (0.003) ***	+ → + → +
Convenience stores	0.092 (0.004) ***	0.067 (0.006) ***	0.050 (0.005) ***	+ → + → +
Schools	0.144 (0.004) ***	0.099 (0.005) ***	0.114 (0.005) ***	+ → + → +
Childcare facilities	0.063 (0.003) ***	0.052 (0.005) ***	0.059 (0.004) ***	+ → + → +
Retail shops	0.036 (0.003) ***	0.042 (0.003) ***	0.015 (0.003) ***	+ → + → +
Welfare facilities for older adults	0.020 (0.003) ***	0.012 (0.004) **	0.005 (0.003) ***	+ → + → +
Hospitals	0.028 (0.005) ***	0.017 (0.006) **	0.021 (0.006) ***	+ → + → +
Pharmacies	0.010 (0.004) **	-0.016 (0.005) ***	0.037 (0.004) ***	+ → − → +
Bus stops	0.029 (0.003) ***	-0.004 (0.005)	0.122 (0.004) ***	+ → · → +
Stations	0.056 (0.005) ***	0.009 (0.006)	0.000 (0.006)	+ → · → ·
Maximum flooding depth (ref: 0.0 m)				
(0.0, 3.0)	-0.122 (0.003) ***	-0.118 (0.005) ***	-0.028 (0.004) ***	− → − → −
[3.0, 5.0)	-0.188 (0.005) ***	-0.248 (0.005) ***	-0.006 (0.005)	− → − → ·
[5.0, inf]	-0.219 (0.007) ***	-0.213 (0.007) ***	-0.085 (0.010) ***	− → − → −
Located inside sediment-hazardous areas	-0.026 (0.005) ***	-0.078 (0.007) ***	-0.008 (0.009)	− → − → ·
AIC^e^	3507.236	1773.203	-8107.784	
Pseudo *R*^2^	0.509	0.543	0.463	
Sample number	71,289	40,661	30,628	

^a^ The years/quarters of transactions, floor space, land area, road width in front of the house, age of the house, main window orientation, travel time to Tokyo Station, and land use zone were adjusted.

^b^ The values in parentheses are standard errors.

^c^ Density was transformed into a nonnegative natural logarithmic scale.

* *p* < 0.05,

** *p* < 0.01,

*** *p* < 0.001.

^d^ Signals (+, −, and ·) correspond to positive, negative, and neutral signals, respectively; the direction (positive, negative, or neutral) was based on a criterion at the significance level of 0.05.

^e^ Akaike information criterion.

Columns 3(b) and (c) show the results for the central and other cities. Most of the variables were related to the sale price, and the results were similar to those for the total sample. However, there were also differences in the results for each subarea. A high intersection density was insignificant in the case of central cities but negatively related to the sale prices of houses in the case of other cities (β = -0.099). A high density of restaurants was positively related to the sale price in the case of central cities (β = 0.048) but was insignificant in the case of other cities. In contrast, a high density of bus stops was positively related to the sale price in the case of other cities (β = 0.122) but was insignificant in the case of central cities. A high density of pharmacies was negatively related to the sale price in the case of central cities (β = -0.016) but positively in the case of other cities (β = 0.037). Flood and sediment risk were negatively related in the case of central cities (β = -0.118, β = -0.248, and β = -0.213 for low to high levels of flooding risk, respectively; β = -0.078 for sediment risk), but moderate flood risk (maximum flooding depth ranging from 3.0 to 5.0 m) and sediment risk were insignificant in the case of other cities.

### Results of regression models by price range

Table 4 summarizes the results of the conditional quantile regression models regarding representative conditional quantiles, which are the (a) 0.15, (b) 0.50 (median), and (c) 0.85 quantiles (Model 2). High net residential density was negatively related to sale price in the 0.85 quantile (β = -0.009), a similar result to that of the model for the median sale price (β = -0.003). However, it was insignificant in the 0.15 quantile. High intersection density was negatively related to sale price in the 0.85 quantile (β = -0.010); however, it was insignificant in the 0.15 and 0.50 quantiles. The facility density and time distance to the nearest station showed a similar trend (with some differences) to the model for the median sale price, except for restaurants (negative relationship of restaurant density with sale price in the 0.15 quantile, β = -0.003) and pharmacies (negative relationship of pharmacy density in the 0.85 quantile, β = -0.029). Single-family detached houses in flood- and sediment-hazardous areas had lower sale prices than those in nonhazardous areas.

**Table 4:** Comparison of the results^a^^,^^b^ of the conditional quantile regression models by price range (N = 71,289).

	(a) 15%	(b) 50%	(c) 85%	Difference in the direction of the coefficient^d^ (a) → (b) → (c)
Higher net residential density^c^	0.001 (0.001)	-0.003 (0.001) ***	-0.009 (0.001) ***	· → − → −
Higher intersection density^c^	-0.006 (0.003)	-0.006 (0.003)	-0.010 (0.004) **	· → · → −
Shorter time distance to the nearest station	-0.010 (0.000) ***	-0.013 (0.000) ***	-0.014 (0.000) ***	− → − → −
Higher facility density^c^				
Bars	-0.068 (0.002) ***	-0.077 (0.003) ***	-0.087 (0.003) ***	− → − → −
Cafés	0.082 (0.003) ***	0.104 (0.003) ***	0.148 (0.004) ***	+ → + → +
Restaurants	-0.001 (0.003)	0.014 (0.004) ***	0.019 (0.005) ***	· → + → +
Hobby classes	0.028 (0.003) ***	0.032 (0.003) ***	0.045 (0.003) ***	+ → + → +
Cultural institutions	-0.040 (0.003) ***	-0.060 (0.003) ***	-0.081 (0.004) ***	− → − → −
Parks	0.057 (0.004) ***	0.052 (0.005) ***	0.047 (0.006) ***	+ → + → +
Gyms and fitness clubs	0.043 (0.003) ***	0.063 (0.003) ***	0.082 (0.003) ***	+ → + → +
Convenience stores	0.108 (0.004) ***	0.092 (0.004) ***	0.094 (0.005) ***	+ → + → +
Schools	0.167 (0.004) ***	0.144 (0.004) ***	0.142 (0.004) ***	+ → + → +
Childcare facilities	0.048 (0.003) ***	0.063 (0.003) ***	0.059 (0.004) ***	+ → + → +
Retail shops	0.033 (0.003) ***	0.036 (0.003) ***	0.035 (0.003) ***	+ → + → +
Welfare facilities for older adults	0.028 (0.003) ***	0.020 (0.003) ***	0.015 (0.003) ***	+ → + → +
Hospitals	0.018 (0.004) ***	0.028 (0.005) ***	0.073 (0.006) ***	+ → + → +
Pharmacies	0.029 (0.004) ***	0.010 (0.004) **	-0.029 (0.005) ***	+ → + → −
Bus stops	0.061 (0.004) ***	0.029 (0.003) ***	0.030 (0.004) ***	+ → + → +
Stations	0.068 (0.005) ***	0.056 (0.005) ***	0.062 (0.006) ***	+ → + → +
Maximum flooding depth (ref: 0.0 m)				
(0.0, 3.0)	-0.078 (0.004) ***	-0.122 (0.003) ***	-0.134 (0.004) ***	− → − → −
[3.0, 5.0)	-0.114 (0.003) ***	-0.188 (0.005) ***	-0.189 (0.006) ***	− → − → −
[5.0, inf]	-0.113 (0.005) ***	-0.219 (0.007) ***	-0.278 (0.016) ***	− → − → −
Located inside sediment-hazardous areas	-0.004 (0.004)	-0.026 (0.005) ***	-0.041 (0.007) ***	· → − → −
AIC^e^	22298.37	3507.236	25155.46	
Pseudo *R*^2^	0.437	0.509	0.531	

^a^ The years/quarters of transactions, floor space, land area, road width in front of the house, age of the house, main window orientation, travel time to Tokyo Station, and land use zone were adjusted.

^b^ The values in brackets are standard errors.

^c^ Density was transformed into a nonnegative natural logarithmic scale.

* *p* < 0.05,

** *p* < 0.01,

*** *p* < 0.001.

^d^ Signals (+, −, and ·) correspond to positive, negative, and neutral signals, respectively; the direction (positive, negative, or neutral) was based on a criterion at the significance level of 0.05.

^e^ Akaike information criterion.

### Results of regression models for each neighborhood characteristic by conditional quantiles

**Fig 3:**
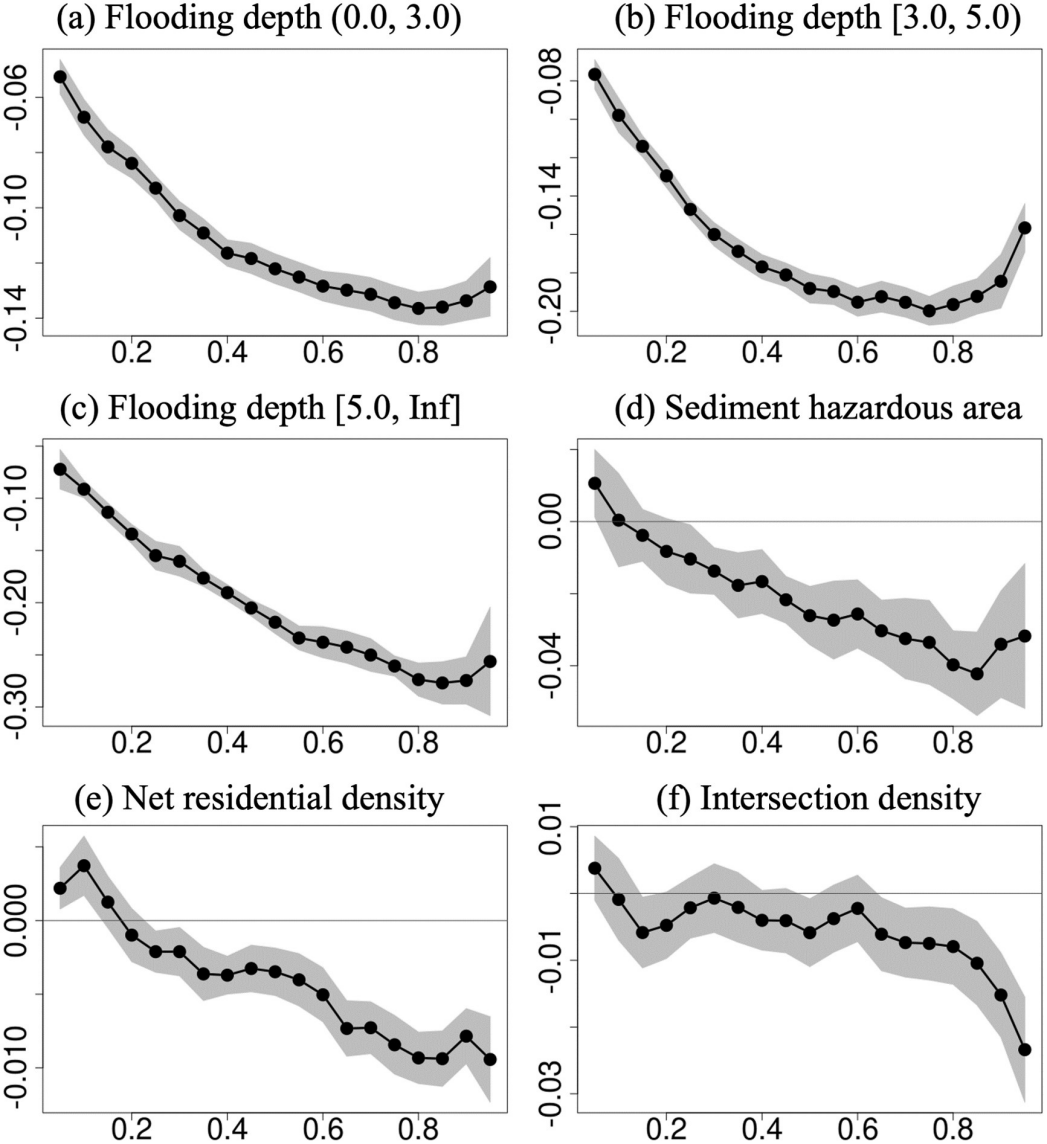
Estimates of the coefficients of flood/sediment risk, net residential density, intersection density, and percentage of older adults for sale price quantiles ranging from 0.05 to 0.95 at 0.05 intervals. The estimates are from the conditional quantile regression model. The years/quarters of transactions, floor space, land area, road width in front of the house, age of the house, main window orientation, travel time to Tokyo Station, and land use zone were adjusted. Conditional quantiles are displayed on the x-axis, and coefficients are presented on the y-axis. The black dots show the estimated coefficients. The gray area represents the 95% confidence intervals.

**Fig 4:**
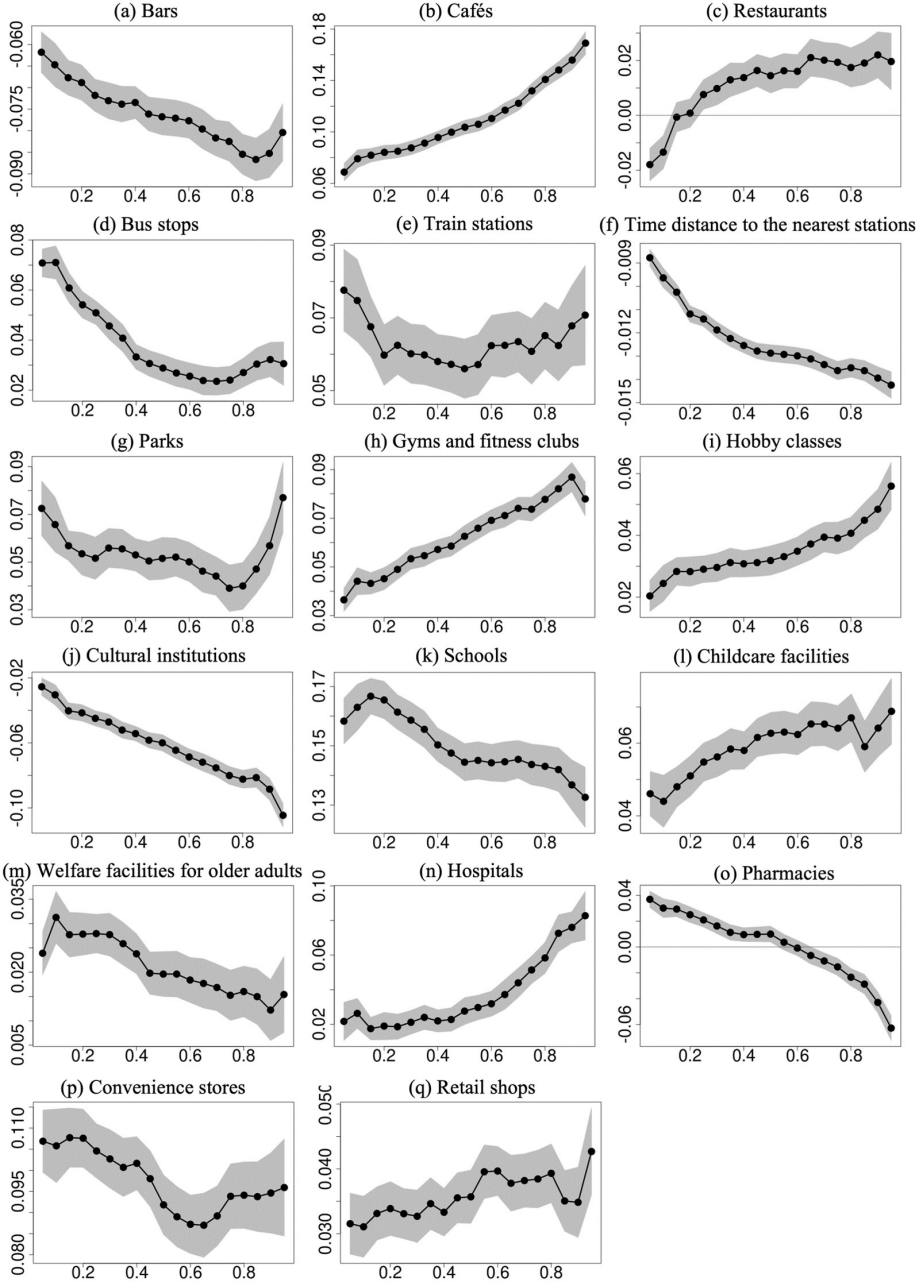
Estimates of the coefficients of facility density for quantiles of sale prices, ranging from 0.05 to 0.95 at 0.05 intervals. The estimates are from the conditional quantile regression model. The years/quarters of transactions, floor space, land area, road width in front of the house, age of the house, main window orientation, travel time to Tokyo Station, and land use zones were adjusted. Conditional quantiles are displayed on the x-axis, and coefficients are presented on the y-axis. The black dots show the estimated coefficients. The gray area represents the 95% confidence intervals.

Figs 3 and 4 present the estimates of the conditional quantile regression models for the conditional quantiles of the sale price, ranging from the 0.05 to the 0.95 quantile at 0.05-point intervals. Fig 3(a)–3(d) show the coefficients for flood and sediment risks. As mentioned in the brief comparison above, being located in a hazardous area was negatively related to the sale price of single-family detached houses in all the quantiles. The higher the quantile, the greater the negative relationship with the sale price for houses in hazardous areas. Interestingly, when the maximum flooding depth was between 3 and 5 m, the coefficient increased slightly in the highest quantile. The coefficient of net residential density was positive in the lower quantiles; however, it became negative in the 0.25 quantile and decreased further in the higher quantiles (Fig 3[e]). In the case of intersection density, its coefficient was negative in the 0.70 quantile and higher but insignificant in the other quantiles (Fig 3[f]).

Fig 4 shows the coefficients of facility density by facility type. Most facility types were positively related to the sale price, but there were also certain further trends or greater, lesser, or similar positive relationships in higher quantiles. It is also true that some facility types indicated a negative relationship in either some or all quantiles. The density of bars and cultural institutions showed a negative relationship with the sale price in all quantiles and a greater negative relationship in higher quantiles. In the case of restaurants, their density was negatively related to the sale price in lower quantiles but became positive in the 0.25 quantile and higher. Conversely, the density of pharmacies was positively related to the sale price in lower quantiles but became negative in the 0.7 quantile and higher.

## Discussion

### Main findings

The Tokyo Metropolitan Area has restricted available land due to geographical constraints; therefore, single-family detached houses have been developed in areas with a flood/sediment risk, not only on plains with high walkability [23]. In Japan, the production of hazard maps by municipalities has been promoted [64], and hazard information has been disclosed to home buyers before transactions take place [65]. This study estimated the relationship between the neighborhood environment (neighborhood walkability and flood/sediment risk) and the sale prices of single-family detached houses. We also compared the relationships between subareas and conditional quantiles and observed differences. The main findings from the comparison in the Japanese context are as follows.

First, there was a difference in preferences between central and noncentral cities; however, the conditional quantiles of the sale price also showed a difference. Second, there were facility types with a difference in the relationship between facility density and sale price by the conditional quantiles of the sale price. As previously mentioned, assuming a budget constraint, those who can pay more for a house with similar features to others (i.e., people who consider buying houses with higher conditional quantiles of the sale price) are more likely to be high-income earners. This suggests that there may be a difference by income level in the preference for neighborhood facilities. Therefore, walking opportunities should be considered within the context of residents’ daily lives, which may differ by socioeconomic status. Third, it was found that people tend to devalue single-family detached houses with a relatively higher sale price in flood/sediment-hazardous areas more. However, for houses in areas with a moderate flood risk (i.e., the maximum flooding depth was between 3 and 5 m), the coefficient was found to increase slightly in the highest quantile. This implies that people who consider buying houses with a relatively higher sale price may devalue a moderate flood risk to a greater degree. Finally, the significance of population and street connectivity was found to vary depending on the price level; they showed a negative relationship with the sale price for houses with a relatively higher price. This implies that external diseconomies should be considered.

### Findings considering differences by centrality

An analysis of different areas could capture the difference by subareas in the relationship between the neighborhood environment and the sale price of single-family detached houses. For example, on the one hand, the difference in the relationship with intersection density may be due to car dependence: Because noncentral cities (suburban and rural areas) have a higher car dependency [55, 56], people may be less inclined to live in areas with high intersection densities that are unpleasant for car drivers, and this may have resulted in a negative relationship with the sale price. On the other hand, the density of bus stops was found to show a positive relationship with the sale price only in noncentral cities (i.e., the car-oriented environment in noncentral cities makes people residing there value houses that are a walkable distance from bus stops more than those living in central cities). The number of restaurants within a walkable distance from each house was found to have a positive relationship only in central cities; this implies that people living in central cities value the walking opportunities close to their homes more than those living in noncentral cities (i.e., the difference between central and noncentral cities may be due to residents’ preference for cars).

Meanwhile, the density of pharmacies was found to exhibit a negative relationship with the sale price of houses in central cities, although a positive relationship was observed in the case of noncentral cities and the target area (all samples). This was in contrast to the case of hospitals, which was found to be positively related to the sale price of houses in both central and noncentral cities. In Japan, pharmacies provide medicines according to the prescriptions issued by hospitals (i.e., the medical services provided by pharmacies depend on hospitals) [81]. Owing to the structure of medicine prescriptions, the benefits from the high density of pharmacies may decrease in central cities where many hospitals are located. Another possible reason for the negative relationship with the sale price of houses in central cities is the limited potential for diversity in neighborhood facilities. Owing to the limited space and number of tenants in densely developed areas, the number of pharmacies within a neighborhood possibly limits the potential for locating other amenities there (e.g., restaurants, food stores, and hospitals). Therefore, people living in central cities devalue the number of pharmacies around their homes; this possibly results in the negative relationship between the density of pharmacies and the sale price of houses. In noncentral cities, pharmacies (including drugstores) provide daily commodities, not only medicines [82]. In other words, pharmacies in noncentral cities provide services that compensate for those provided by supermarkets; therefore, people living in noncentral cities value pharmacies near their homes [83].

As regards disaster risk, some variables in noncentral cities were not found to show a significant relationship with sale price. People living in central cities have the option of houses at lower prices located in areas with flood risk. Meanwhile, there are few plains with a low risk of flooding in the suburbs, so suburban dwellers (especially those with a low income) may not have been able to lower the prices of their houses significantly to reflect the risks.

### Findings considering differences by price range

For most facility types, the number of walking opportunities within walking distance from houses was found to be positively related to the sale price of single-family detached houses in all quantiles; this is in line with findings from outside Japan [13, 14, 46, 84] suggesting that a close distance to amenities increases property value. However, the results also show that this relationship does not hold true for bars, restaurants, pharmacies, or cultural institutions. The densities of bars and cultural institutions were found to be negatively related to sale price in the lower quantiles, and this negative relationship was stronger in the higher quantiles. These facilities are destinations not only for residents of the neighborhood but also for visitors from other areas beyond walking distance, which may cause congestion and noise issues, and these facilities may be visited less frequently. This could be the reason for the negative relationship with sale price given that people prefer calm, low-traffic residential areas [85, 86]. The findings suggest that sufficient consideration of these issues is important for policymakers discussing the development of safe and walkable neighborhoods.

In addition, as the price level differs, changes were observed in the relationship between the facility density of restaurants and pharmacies and sale price. The density of restaurants and pharmacies was found to change from negative to positive and from positive to negative, respectively, in relation to sale price as the quantile increases. As previously acknowledged, those who can pay more for a house with similar features are more likely to be high-income earners, given their budget flexibility. Residents’ preference for a high density of pharmacies diminishes as the quantile of the sale price increases and tends to be greater in the higher quantiles (in contrast to the case of hospitals, which are positively related). Similarly to the case of the negative relationship between the density of pharmacies and the sale price of houses located in central cities, the number of pharmacies within a neighborhood possibly limits the potential for locating other amenities in the neighborhood. Although the number of pharmacies provides benefits for those who consider buying houses with a relatively lower price, high-income earners possibly devalue houses with limited potential for other amenities (e.g., restaurants) in their neighborhood. Indeed, high-income people tend to visit restaurants frequently [87, 88]. Full-service restaurants are more likely to be located in neighborhoods with high socioeconomic status owing to the high demand of high-income earners living in such areas [89, 90], and they provide a healthier and more diverse eating environment for the residents [91, 92], who thus benefit from access to a number of restaurants within walking distance. In contrast, low-income people may perceive disparity between themselves and restaurant visitors in daily life; thus, they do not prefer areas with a high density of restaurants. Further studies are necessary to explore the reasons for the variation in the relationship between sale price and neighborhood facilities.

Single-family detached houses located in areas with a high risk of flooding and sediment were found to be traded at a lower price in all quantiles. People tend to devalue single-family detached houses with relatively higher sale prices in flood- and sediment-hazardous areas more. Findings from outside Japan [41, 43] suggest that the risk discount in the sale price could be affected by home buyers’ risk awareness. This implies that high-income earners are more likely to be aware of the flood/sediment risk, and they reflect the risk awareness in the sale price. Conversely, it was observed that the negative relationship was slightly mitigated in the highest quantile of sale prices for houses in areas with a moderate flood risk; this is in line with findings from England, the United Kingdom [36] and Taipei, Taiwan [40] (where, similarly to the Tokyo Metropolitan Area, available land is limited and flooding is frequent). Houses in flood-hazardous areas can also be viewed as those located close to waterfront areas [93, 94], and this proximity increases their value by providing easily accessible places for daily leisure and recreational activities [95, 96]. In addition, dense development in waterfront areas, such as high-rise residential and commercial buildings, possibly causes prices to increase. These factors may be attributable to the mitigation observed in the highest quantile. However, in high-risk cases, where the second and higher floors may be inundated, sufficient mitigation for the negative impact of flood risk cannot be expected; in other words, the high risk of flooding leads people to ascribe less value to the benefits of riverside amenities near their homes. Therefore, policymakers should discuss ways to diminish damage due to natural disasters and maximize the benefits from the natural environment, along with waterfront development, to make the riverside areas safe and amenable.

High net residential density and intersection density were found to be negatively related to sale price in the higher quantiles and to be positive and insignificant in the lower quantiles, respectively. High residential density means high potential to provide and maintain walking opportunities within a neighborhood. A grid-patterned road network (an area with high intersection density) enables people to choose from a variety of route options and to travel to a destination via a shorter distance than a curvilinear pattern, which facilitates frequent walking [17, 97]. In contrast, for single-family detached houses with relatively high sale prices, there could be external diseconomies of congestion and noise caused by high net residential density, and the potential to maintain a high level of urban services owing to the dense population might also decrease. High-income earners may place more value on less congestion around their homes [98]; therefore, high population density was negatively related to the sale price in the higher quantiles. A very high intersection density indicates a small block size, where the area of floor space may be restricted. People who consider buying houses with a relatively higher price may reflect the potential for a restricted area of floor space in the sale price. Indeed, areas with a very high intersection density require rezoning to improve land efficiency, such as combining some blocks, which decreases the intersection density. Moreover, in areas with the highest level of population and intersection density, the higher density does not increase residents’ walkability [99]; indeed, the extremely high density lowers their step counts [100]. This implies that there could be an adequate level of population and intersection density for both walking behavior and the sale prices of single-family detached houses.

A safe and walkable neighborhood is a place where creative people gather [52, 53]. In other words, a safe and walkable neighborhood may attract a certain class of people by making it unaffordable for others to live in the area [101]. In addition, some low-income people may have no choice but to buy houses in disaster-hazardous areas because they have few options for affordable houses within their limited budget. Therefore, flood/sediment prevention efforts in hazardous areas are necessary to provide residents with safe and walkable neighborhoods. The findings from this study suggest that the preference for a safe and walkable neighborhood is not homogeneous and differs by socioeconomic status (inferred from the sale prices of houses) in addition to region. The difference in preference based on income level can lead to inequities in walkability and safety between high- and low-income neighborhoods (indeed, low-income neighborhoods have fewer amenities and lower levels of safety [90]). This implies that simply constructing densely developed areas with a variety of amenities is not the ultimate solution for creating a safe and walkable neighborhood, irrespective of income level. Therefore, it is important to combine this with creating a socially inclusive environment; providing affordable houses located in safe and walkable neighborhoods may be an option to consider. Policymakers should consider the use of indices for assessing the neighborhood environment according to the target socioeconomic status and discuss ways to develop neighborhoods that are attractive to people from diverse backgrounds, with careful consideration of income inequity.

### Limitations

This study focused on the sale prices of single-family detached houses in the Tokyo Metropolitan Area and their relationship with the neighborhood environment to capture differences in the relationship among conditional quantiles of sale prices. However, it has several limitations. The study only considered income level, inferred from the sale prices of houses, as an index of socioeconomic status. However, people’s preference regarding neighborhood environment may also differ by other sociodemographic factors, such as gender, age, education level, ethnicity, and relationships with family members [102]. Owing to privacy issues, these individual characteristics of those buying houses have not been recorded. Further data collection should be conducted to clarify the related difference in the preference for neighborhood environments while studying sale price. We considered the neighborhood walkability, flood and sediment risk, and percentage of older adults as neighborhood-level variables. Other possibly relevant neighborhood-level variables, such as socioeconomic neighborhood characteristics, neighborhood scenery, and social networks in neighborhoods, were not considered. These may be confounding or mediating variables. Another limitation pertains to causal effects. Finally, the index of facility density, which was used to estimate neighborhood walking opportunities, did not include the service quality or attractiveness of each facility, which may cause the extent of each facility’s impact to vary. There is also a modifiable area unit problem [103]. Further studies considering these issues are necessary to clarify the scale of development that can best improve neighborhood walkability and foster effective neighborhood development.

## Conclusion

This study estimated the relationship of single-family detached house prices with neighborhood walkability and disaster risk and clarified the differences by sale price using conditional quantile regressions. The results showed differences in the relationship between the sale price and the facility density of each type within a walking distance based on the price range. For most facility types, the number of walking opportunities within walking distance from houses was found to be positively related to the sale price of single-family detached houses in all quantiles. However, it was also found that this relationship did not hold true for bars, restaurants, pharmacies, or cultural institutions. A high net residential density and high intersection density were negatively related to the sale price in the higher price range but positively and insignificantly related to it in the lower price range, respectively. Flood and sediment disaster risk were negatively related to sale price, especially in the higher price range. Meanwhile, it was also found that the negative relationship was slightly mitigated in the highest quantile of sale prices for houses in areas with a moderate flood risk (maximum flooding depth: 3–5 m). In cities with geographic constraints (e.g., an island with many mountains close to the sea and rivers), there could be a trade-off between flood risk and neighborhood walkability owing to their restricted available land. The findings suggest that preferences for neighborhood walkability and safety vary according to people’s income levels inferred from the sale prices of houses. When policymakers assess neighborhood environment, indices reflecting the target socioeconomic status should be used to consider diverse preferences.
